# Bilateral breast carcinoma– a rare case report

**Published:** 2011-02-25

**Authors:** AK Dalal, A Gupta, R Singal, U Dalal, AK Attri, P Jain, A Sharma, S Gupta

**Affiliations:** * Department of Surgery–Government Medical College and Hospital, Sector–32, Chandigarh, PunjabIndia; ** Department of Anatomy–Adesh Institute of Medical Science and Research, Bathinda, PunjabIndia; *** Department of Surgery, Maharishi Markendeshwar Institute of Medical Sciences and Research,Mullana (Distt–Ambala) Haryana India; **** Department of Medicine, Maharishi Markendeshwar Institute of Medical Sciences and Research, Mullana (Distt–Ambala) HaryanaIndia; ***** Department of Radiodiagnosis and imaging, Maharishi Markandeshwar Institute of Medical Sciences and Research, Mullana, (Distt–Ambala) HaryanaIndia

**Keywords:** synchronous, carcinoma, breast, ultrasonography, mastectomy, chemotherapy

## Abstract

We are presenting a rare case of bilateral breast cancer. A 65–year–old female reported to us with complaint of lump in the left breast. There was positive family history. The patient was diagnosed with carcinoma breast of left side on edge biopsy and of right side by ultrasonography, which was confirmed on fine needle aspiration cytology. At the time of admission, ultrasonography of the abdomen and right breast were normal. After 1 month, the patient was readmitted with redness over the inner quadrant of the right breast skin

## Introduction

A second primary breast cancer in the opposite breast can be either synchronous or metachronous. The majority are metachronous [1]. Bilateral synchronous breast cancer accounts for 0.2–2% of all breast cancers [2]. Synchronous bilateral breast carcinoma (SBBC) is an uncommon presentation, and the management of patients with this disease is not well established [3]. Breast cancer is usually associated with local and lymphatic spread and with blood–borne spread to lungs, bones and liver. Our patient's bilateral breast carcinoma (BBC) was consistent with this definition of being SBBC.

Case report–A 65 year–old woman came to our Breast unit due to a palpable lesion in her left breast. From the personal history, the woman had risk factors of positive family history for breast cancer. Her mother was diagnosed with breast cancer of right side. There were no other complaints like weight loss, fever or loss of appetite.

Clinical examination of the left breast revealed a hard lump in the upper quadrant of size 14 x 10 cm that was fixed to the underlying structures. The overlying skin of the breast along with nipple and areola were involved. Multiple lymph nodes were felt in the axilla, out of which one was large in size–of about 2x3cm, which was mobile. 

**Figure 1:**
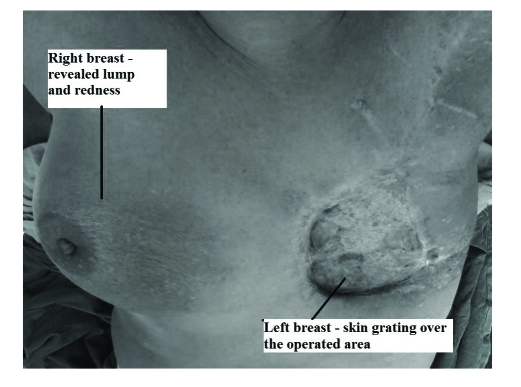
Gross picture showing operated site on left breast and redness over the right breast region

On right side of breast, skin was reddish in colour in lower and inner quadrant but no lump was felt (Figure 1). Nipple was inverted. No axillary lymph nodes were felt. Routine blood tests were within normal limits including chest X–ray.

In ultrasound of the left breast, the consistency of the lesion was solid and multilobular. Additionally, ultrasonography of the right breast demonstrated a non–palpable lesion, consisting of clustered micro calcifications of a diameter equal to 0.7 cm in the upper outer quadrant. Abdominal ultrasonography revealed no metastasis. Fine needle aspiration cytology confirmed the malignancy of both breasts. On the right side, it came as invasive lobular carcinoma and on the left side, it came as ductal carcinoma. Modified radical mastectomy with axillary clearance and skin grafting was performed on the left breast. Pathological specimen showed a large well-demarcated tumour mass measuring 14 x 10 x 9 cm, firm to hard in consistency. Histopathological examination confirmed the diagnose of ductal carcinoma with lymph node infiltration. Specimen revealed 9 lymph nodes. Post–operatively, the patient was on adjuvant chemotherapy and responded very well to the treatment. 

## Discussion

Synchronous tumours are defined as two or more tumours where each are malignant, are distinct from each other i.e. of different histological type and where neither can originate with metastasis from another tumor [4]. The incidence of synchronous bilateral cancer is of approximately 1% to 2% and that of metachronous cancer 5% to 6%.Bilateral synchronous breast cancer is an uncommon finding in women presenting with multiple breast lumps. It is reported to account for approximately 1% to 2% of women with breast cancer whereas metachronous breast tumours account for 5% to 6% of cancer cases [5]. The cancer can be invasive or noninvasive [1]. The incidence pattern of synchronous cancer is similar to that of unilateral disease, although without any notable trends in recent decades [6]. Although its aetiology is not well understood, however it appears that this familial link is more likely with metachronous bilateral breast cancer than either unilateral or synchronous bilateral cases. Furthermore, the risk of having breast cancer is substantially increased with a first–degree relative with bilateral breast cancer [7]. Such lower disease free survival and high rates of distant metastasis is a recognized feature of bilateral synchronous tumors, which therefore have a worse overall survival compared to unilateral tumours [5]. However, there does not appear to be any difference in survival if synchronous tumours are compared to the metachronous ones. The gradual increase in the incidence of synchronous disease during the 1970s coincides with the introduction of routine and bilateral mammography as part of the diagnostic work-up in women with unilateral cancer [6]. Bilateral cancers are detected early by preclinical work–up, and classified as synchronous disease rather than diagnosed later as metachronous disease [6]. Synchronous breast cancer has a poorer prognosis than metachronous or unilateral breast cancer [8]. Nowadays, the incidence of local recurrences, bilateral cancer, and distant metastasis are reduced with the use of adjuvant systemic therapy, mainly tamoxifen and chemotherapy that became clinical practice [9].

## Conclusion

The incidence of invasive cancer detected by random biopsy of the opposite breast is not high enough to justify routine adoption of this procedure. The remaining breast must be followed for the remainder of the patient life by physical examination and annual mammography. The treatment of the secondary primary breast cancer should be that appropriate for the stage of the disease. The prognosis for the woman with a second primary breast cancer is quite favorable and is dependent on the stage of both the first and the second cancer. Source of funds and conflict of interest is nil.
